# Dynamic Quantum Operations at Elevated Temperatures Using Hot-Spot Nanoheating of Color Centers

**DOI:** 10.1021/acs.nanolett.5c04008

**Published:** 2025-08-28

**Authors:** Frank D. Bello, Daniel D. A. Clarke, Daniel Wigger, Ortwin Hess

**Affiliations:** † School of Physics and CRANN, 8809 Trinity College Dublin, Dublin 2, Ireland; § Advanced Materials and Bioengineering Research (AMBER) Center, 8809 Trinity College Dublin, Dublin 2, Ireland

**Keywords:** near-field transducer, plasmonic nanoheating, quantum control, entanglement, photon pairs, color centers

## Abstract

Temperature fluctuations in materials used for quantum networks can give rise to lattice vibrations that detune, dephase, and decrease the lifetimes of embedded quantum defects that function as qubits. Most experiments demonstrating quantum operations have been performed at cryogenic temperatures ranging from milli- to a few kelvin, thereby reducing these adverse effects. However, encouraged by the relatively long lifetimes recently discovered for group IV color centers, we aim to show that subdiffracted heating, i.e., nanoscale thermal “hot spots”, produced by a plasmonic transducer can control the resonant behavior of individual qubits. Our analysis, reported over largely unexplored physical dimensions for the nanoheating of qubits, establishes the ability to perform dynamic quantum operations at elevated temperatures via thermally mediated control of two-photon coherence and subsequent photon-number entanglement. As such, color centers raise encouraging prospects for advancing on-chip quantum photonics and elevating solid-state quantum information processing technologies toward higher temperatures.

## Graphical abstract

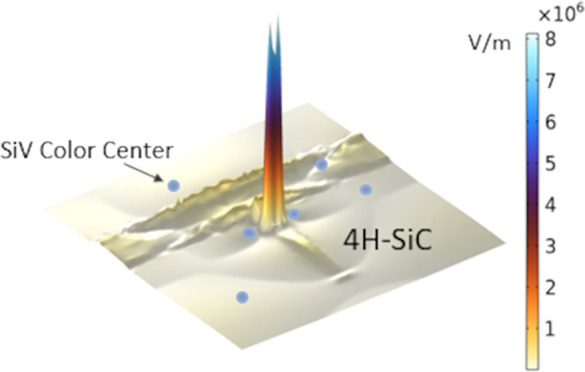

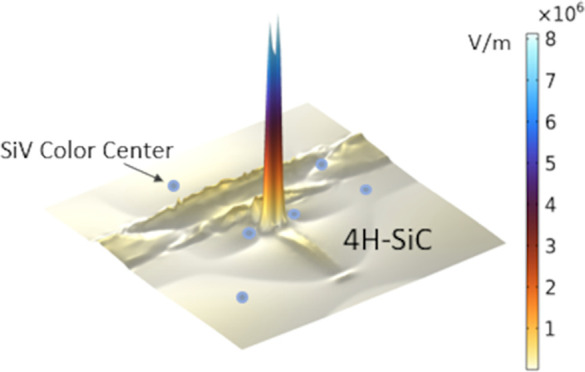

There are a handful of qubit architectures available for providing a steady source of entangled quantum states to use within quantum networks, for instance, superconducting transmon qubits,^1,2^ trapped ions,^3^ or photons.^4–6^ Underlying these architectures is their sensitivity to decoherence when quantum operations are performed at elevated temperatures. Our research aims to develop a platform that raises the temperatures used for quantum operations as well as ease the fabrication constraints on quantum information devices making them more scalable for manufacturing, a significant challenge for multiqubit systems. For this, we focus on using optically emitting vacancy centers (VCs), a type of color center, as qubits and a source of entangled photon states. Such VCs, in wide-bandgap crystals, like diamond or silicon carbide,^7^ are deemed beneficial with charge and spin state lifetimes well over a nanosecond, along with their ability to facilitate quantum-device functionality at room temperature, such as quantum memories.^8,9^ More recently, plasmonically coupled quantum emitters are also seen as a viable pathway for moving quantum networks toward higher temperatures given the high speed of light–matter interactions relative to decoherence rates within plasmonic structures^10^ along with their reported strong coupling at room temperature.^11,12^ A wide variety of plasmonic resonator designs have been explored for their ability to confine light many times below the diffraction limit by coupling photons to shorter wavelength surface plasmons.^13,14^ Therefore, within a modeled experiment, we couple the subdiffracted light of a plasmon mode to two nearby vacancy centers used as qubits for the dynamic generation of photon-number, i.e., number-state, entanglement. Using this theoretical framework, we pursue two goals: (1) to exploit strong-coupling behavior for coherent manipulation of qubit states and (2) to utilize Joule nanoheating via the plasmonic near field to induce lattice vibrations, i.e., phonon occupation, that shift the transition energies of the individual qubits in and out of resonance with the near field for performing quantum operations. Thus, as the temperature changes over time, it introduces a dynamical switch for optical quantum control. To accomplish this, even under conditions of tens of kelvin, would be a substantial benchmark and would significantly reduce experimental constraints.

As our choice of resonator, we used a near-field transducer (NFT), which is fully integrated with a single-mode photonic waveguide and is independent from the media containing the qubits. This is advantageous for it confers the ability to raster (move independently) above the medium, thus providing nanoscale control over the distance between resonator and qubit architecture,^15^ allowing it to function as a movable plasmonic waveguide with spin-stand capability, similar to state-of-the-art hard drive technologies.^16^ This enables one to couple to and manipulate the temperature change desired for each selected qubit and effectually adjust the transition energies of each, i.e., resonance behavior, faster than dephasing can have a substantially detrimental effect. Subdiffractive, focused nanoheating, i.e., “hot-spot” nanotechnology, has been leveraged with success in several applications, most notably, heat-assisted magnetic recording (HAMR),^17,18^ as well as proposed for directional manipulation of electric currents in ultrathin films.^19^ Here, we demonstrate a level of control enabling the generation of time-averaged entanglement stemming from a two-photon source that is compatible with time-repetition, error-correction experiments and does so at temperatures of many tens of kelvin, vastly higher temperatures compared to current technologies.^1^ Our NFT is designed specifically for nanoheating that achieves changes in the temperature on the order of 10^1^–10^2^ K over areas of just a few tens of nm^2^ (see Figure 1 for details),^16,20^ yielding temperature gradients of 10–20 K/nm. This is comparable to similar state-of-the-art HAMR devices,^21^ where hot spot regions are typically confined to areas of 50 nm^2^.^14,22^ Thus, we can manipulate transitions of the nanoscale two-emitter system, without affecting other nearby VCs.

**Figure 1:**
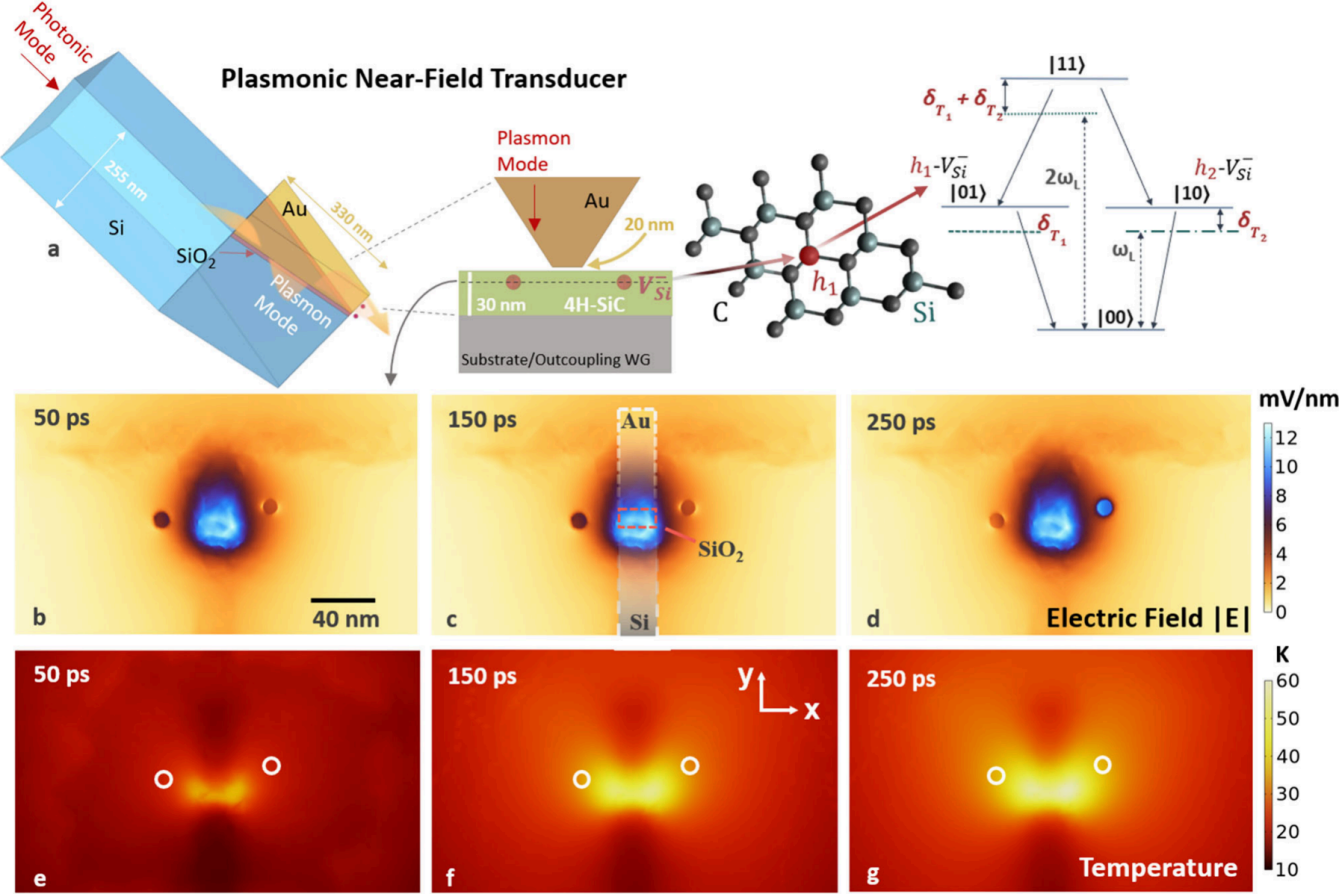
Plasmonic nanoheating of Si vacancy centers. (a) To-scale minimal model of the integrated single-mode photonic waveguide (WG) that evanescently couples to a metal–insulator–semiconductor (MIS, Au–SiO_2_–Si) near-field transducer (NFT) to subdiffract light and create heat on the nanoscale within nearby qubit media (see the Supporting Information for to-scale model with complete dimensions, operational details, and material parameters). A smaller inset depicts the arrangement of negatively charged silicon vacancy centers (*V*_Si_^–^) below the 20 nm wide tip of the NFT that can be fabricated via layer deposition and etching. The substrate is composed of SiO_2_, but it can be replaced with silicon nitride or other material, in addition to a grating coupler, as preferred for outcoupling. The vacancy centers are located at hexagonal lattice sites, *h*_1_ and *h*_2_, of the crystal shown for the 4H polytype of SiC (4H-SiC). The schematic representation on the far right illustrates the four-level bipartite system consisting of the vacancy centers, with individual excited states of each qubit represented as |01⟩ and |10⟩, as well as the state where both vacancies are excited, |11⟩. Detunings (δ_*T*_*i*__) of each state are a function of the temperature, which can be manipulated via input power, *V*_Si_^–^ positioning, and/or NFT rastering. Arrows depict decay processes as well as resonant laser excitation (at a frequency of ω_L_). Panels b–d are planar cross sections (dotted horizontal line in 4H-SiC) through the *V*_Si_^–^ sites (dark/light circles in panels b–g) showing the plasmon-mediated electric near field of the NFT, capable of strongly driving qubits, at various times after an incident field of 0.5 mW is applied. The *c* axis is oriented out of plane. The Joule heating, i.e., power dissipation, from the field produces the nanoscale heating demonstrated in panels e–g, used to control the temperature-dependent detunings and, hence, entanglement. Panel c contains an outline of the NFT surface nearest the 4H-SiC, showing its position relative to the field distribution.

Furthermore, we emphasize the use of silicon vacancy (*V*_Si_^–^) centers as qubits, which have shown considerable promise given their slow radiative decay near room temperature.^23^ This is crucial if we are to produce enough heating to induce optical resonances in the qubits, which can take many tens of picoseconds. Silicon VCs embedded within the 4H polytype of silicon carbide (4H-SiC)^24^ are of particular interest compared to those embedded in a diamond given the relatively stronger absorption of optical energy by the host medium, i.e., Joule heating, but also due to their near-infrared resonant wavelength, useful for optical networks, along with the feasibility of strong, cavity-enhanced optical emission (∼50%) predicted at the zero-phonon line (ZPL) between 861 and 862 nm.^25^ We analyze how controlled heating that adjusts temperatures between roughly 10 and 40 K and up to 80 K impacts quantum properties of *V*_Si_^–^ centers, most notably through energy tuning and temperature-dependent dephasing.^26^

Figure 1a depicts the system architecture that we consider for inducing photon-number entanglement by using nanoscale heating. Near-field energy deposition from the antenna-based NFT, which is manufacturable using thin-film deposition and lithographic processes,^14^ is capable of nanolocalized temperature rises on the order of 10^2^ K, effectively tuning transitions of the *V*_Si_^–^ centers several nanometers in wavelength (≈1–2 THz).^27^ It is designed to work with roughly 5–10% efficiency^16^ between the wavelengths of 810–870 nm and is evanescently coupled to a photonic waveguide via extension of the Si substrate layer (see the Supporting Information for full details/parameters). Herein, the operating wavelength is set to 866 nm, approximately 5 nm (≈2 THz) from resonance with a stable ZPL (V1) line of two *V*_Si_^–^ centers located at hexagonal sites, *h*_1_ and *h*_2_, of the 4H-SiC. The optically addressable states of this pair of *V*_Si_^–^ centers constitute a bipartite system, depicted on the right-hand side of Figure 1a, that serves as a two-qubit register. For clarity, we use the generalized qubit notation, |0⟩ and |1⟩, for each emitter as well as the entangled quantum state post-emission, since they are intrinsically linked. The VCs, which for simplicity are assumed to have equal initial ZPL energies and have optical dipoles oriented along the *c* axis of the SiC crystal, are positioned such that they experience similar but not identical local heating conditions; our aim is to exploit the differential, phonon-mediated shifts in ZPL transition energies for dynamic quantum control purposes (see the Supporting Information for model details).^28–30^

The electromagnetic field distributions in the defect-hosting 4H-SiC film (30 nm thickness), along with the temperature distributions arising from subsequent resistive, i.e. Joule, heating, are displayed for a planar cross section through the center of the two-qubit solid-state architecture (Figure 1b–g), which is located roughly 5 nm from the output end of the NFT.^16^ The 4H-SiC, which is placed on top of a silicon substrate, contains the *V*_Si_^–^ centers (outlined in white/black) that are analyzed for the emission of entangled photons. Figure 1b–d shows the localized, high-intensity electric field at different times during steady-state operation of the NFT. The electric field strength is shown to be roughly 2–3 orders of magnitude larger over an area that is tens of nm^2^ compared to the surrounding region. The vacancy centers themselves separately interact with the near field and may further adjust the local field strength given its interplay with their polarization, as can be seen in Figure 1d, although we note that the nanolocalized electric field and subsequent strong coupling dominate the interaction in the Hamiltonian (see eqs S1–S3 in the Supporting Information). The heating effect due to the subdiffracted light is demonstrated in Figure 1e–g, with nanoscale shaping of the temperature distribution facilitated via the input power and the spatial profile of the plasmonic near field, effectively demonstrating light-controlled nanoheating of the structure. Our analysis investigates *V*_Si_^–^ centers that experience cryogenic (initial) temperatures ranging between 10 and 50 K, beyond which the pure temperature-dependent dephasing that we adopt is expected to shift toward a cubic temperature dependence with the appearance of multiphonon scattering processes in 4H-SiC.^26^

When formulating entanglement, we make use of simultaneously emitted photon pairs, for which we are able to produce notable coherence in the two-photon transition, |00⟩ ↔ |11⟩.^31^ Expected decoherence within our bipartite system at significantly elevated temperatures is anticipated to render little to no measurable entanglement between its aforementioned states, an issue further compounded by the presence of spontaneous decay from the single-qubit, i.e., single-excitation, states |01⟩ and |10⟩. Nevertheless, improved capabilities for filtering photon pairs,^32^ along with quantum state tomography,^33,34^ facilitate the acquisition of a reduced two-photon density matrix, from which the degree of entanglement can be quantified. Furthermore, recent experimental advancements have demonstrated photon-number entanglement that accesses the lesser used vacuum state.^34,35^ In our present approach, we model an idealized, extrinsic filtering process for reconstructing the two-photon density matrix, analogous to that performed in biexciton cascade experiments,^36,37^ which excludes contributions from single-excitation states. We thereby ascertain the degree of photon-number entanglement between state |11⟩ and vacuum state |00⟩ (see the Supporting Information for details). By distilling the two-photon transition, only four non-vanishing elements contribute to the concurrence (i.e., the entanglement measure), consisting of the populations of states |00⟩ and |11⟩ and their coherence. The concurrence is then proportional to the non-local coherence produced, calculated here as a normalized (*N*) time-averaged quantity, $C\left(t\right)=\frac{1}{t}\int_{0}^{t}d_{\tau }2|\rho_{00,11}^{N}\left(\tau \right)|$ .^38^

Values provided for concurrence range between 0 (no entanglement) and 1 (maximum entanglement) for cryogenic temperatures (*T*_in_) set at 10, 30, or 50 K, as shown in Figure 2.^38,39^ As soon as the detuned, optical driving of the NFT is initiated, the 4H-SiC heats up, which shifts the transition energies of the emitters toward the plasmon resonance, illustrated therein by the time-dependent detunings δ_*T*_1__ and δ_*T*_2__.^27^ When the resonance condition is reached, i.e., δ_*T*_1__ and δ_*T*_2__ → 0 (gray dotted line), the two-photon transition is driven more efficiently, resulting in higher concurrence. We observe a decrease in temperature-dependent dephasing when using lower cryogenic, i.e., initial, temperatures, illustrated as we move to the right of the figure (purple → yellow → red curve) with concurrence values more slowly attenuating toward zero. Nevertheless, the achieved concurrence values are comparable, regardless of the initial temperature, when the VCs are more rapidly brought into resonance with the plasmonic mode. This is done via the thermally enabled control of the detuning rate for each VC, whose transition energies can be modulated at an effectively higher rate by either increasing the cryogenic temperature and/or via the input power (*P*_in_), as highlighted in Figure 2b–d. Hence, radiative decay (lifetime of 1 ns) and dephasing (10^1^–10^2^ ps) will happen on longer time scales than the nanoheating required to attain resonance. This is distinctly noticed by comparing concurrence values when using an identical input power, *P*_in_ = 0.5 mW, for different cryogenic temperatures initially set at *T*_in_ = 50 K (purple curve) and *T*_in_ = 30 K (maroon curve), on the far left of the figure. Both scenarios generate time-averaged values (black curves) between 0.68 and 0.72 for concurrence; however, the resonance condition is achieved over a shorter time scale for *T*_in_ = 50 K compared to the lower temperatures, and perhaps counterintuitively, an excellent value (>0.9) for concurrence can be achieved at the elevated temperature. We note that a temperature-dependent detuning between *V*_Si_^–^ centers, i.e., the difference between δ_*T*_1__ and δ_*T*_2__ at each emitter site, is more evident for scenarios with a slower temperature rise, i.e., *T*_in_ = 10 K and *P*_in_ = 0.5 mW, as depicted by the pink and red dashed curves, respectively. This is due to a timing mismatch between VC resonances (≈22 ps), where a detuning of roughly 3.5 THz between ZPL energies yields concurrence values above 0.8. One must take care here as the separation in time when each qubit is brought into resonance must be sufficiently less than the time required for significant decay and dephasing. Figure 2e–g shows the temperature-dependent dephasing rate, which increases most rapidly above 50 K due to multiphonon scattering effects that dominate with a cubic dependence on the temperature.

**Figure 2:**
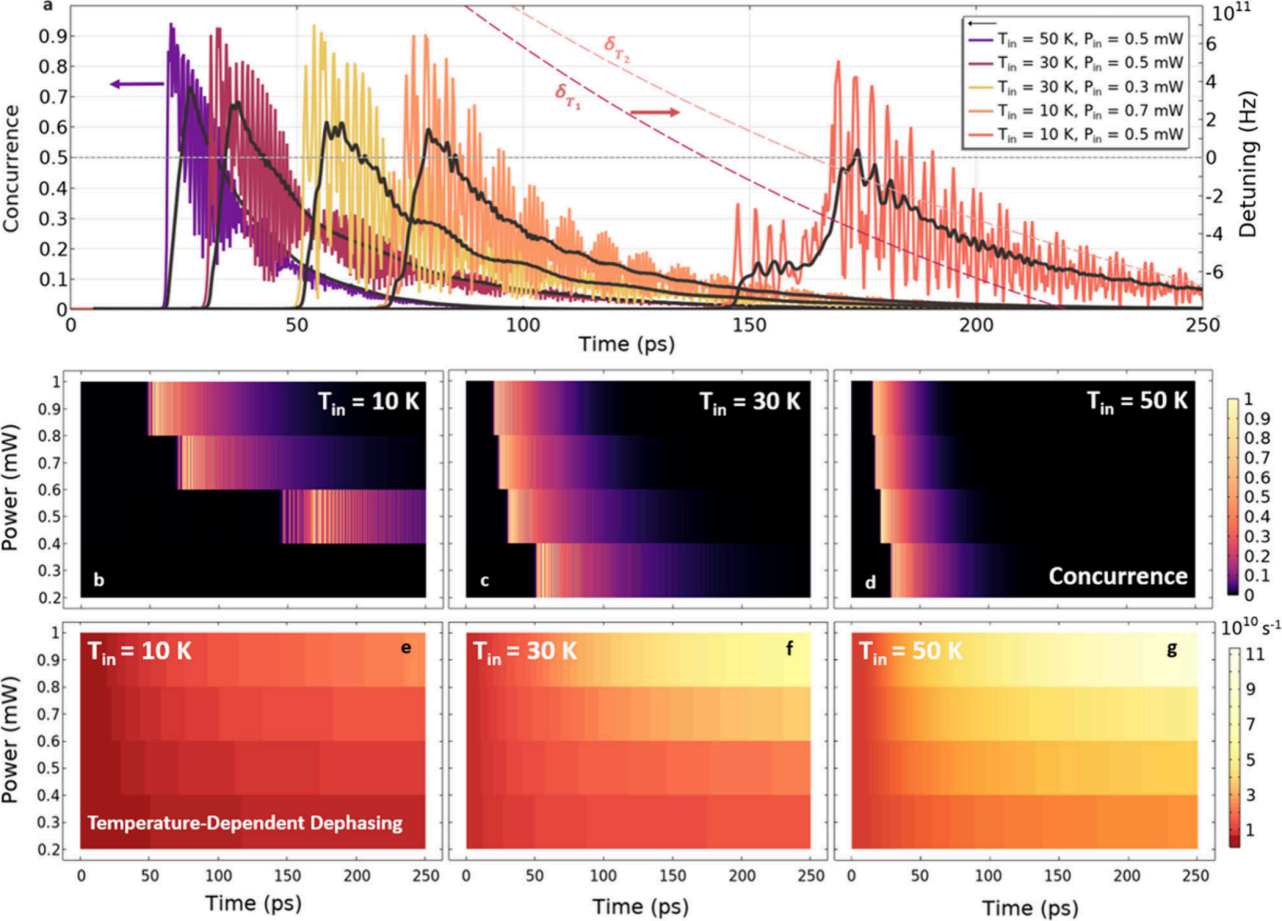
Manipulation of time-dependent concurrence. (a) Concurrence of simultaneously emitted photons is shown for various input powers (*P*_in_) and initial cryogenic temperatures (*T*_in_). By manipulating the input power and, therefore, heating rate of the system, one can induce concurrence by tuning *V*_Si_^–^ centers (VCs) into resonance with the incident near-field well before significant temperature-dependent dephasing sets in. The black curves are the time-averaged concurrence (every 5 ps) for each case presented. When bringing VCs from lower cryogenic temperatures into resonance with the NFT mode, a larger temperature change is required and, hence, a longer time is needed for heating. This is evident when comparing the far-right and far-left curves, which depict concurrence for *T*_in_ = 10 and 50 K, respectively. If the VCs are under varying heat conditions, then the discrepancy in temperature produced at each VC site will tune them into resonance at different times. We see this when plotting the detuning of each VC (δ_*T*_1__ and δ_*T*_2__) coming into resonance (horizontal dotted line) separated by roughly 22 ps (pink/red dashed curves) for the case with *P*_in_ = 0.5 mW and *T*_in_ = 10 K. In panels b–d, the profiles illustrate the nonlinear relationship between heating and concurrence. It is noteworthy that entangled photons with concurrence over 0.5 are repeatedly emitted over tens of picoseconds. We emphasize the case for *P*_in_ = 0.3 mW and *T*_in_ = 10 K, where no entanglement is achieved since the amount of heating applied could not shift the transition energies of the VCs into resonance with the plasmon. The transition dipole moment of each VC is taken to be 5 D (see the Supporting Information for the effect of varying dipole moments). Panels e–g show the temperature-dependent dephasing as a function of input power depicting its effect on concurrence. As rates increase from 10^10^ to 10^11^ s^–1^ (panel g and top of panel f), largely due to multiphonon scattering above 50 K, concurrence values quickly tend to zero.

We further elucidate the distinct possibility of performing dynamic two-qubit operations via nanoheating using a single excitation pulse, such as a controlled NOT (CNOT) logic gate that effectively manipulates the population of qubit states and generates entanglement for use in quantum algorithms post-emission.^40^ As shown in Figure 3c, by bringing one of the VCs into resonance initially, substantial control of its population dynamics can be maintained with time-dependent oscillations in the populations of a single qubit and bipartite state. The schematic in Figure 3a depicts the desired steps for completing a CNOT operation with the two-qubit register. Figure 3b highlights outcomes for each step by presenting the populations and coherences in the bipartite system for the case of *T*_in_ = 10 K and *P*_in_ = 0.5 mW (far-right curve of Figure 2a). Figure 3c shows the controlled excitation of the first VC, i.e., maximized population of state |01⟩ (ρ_01,01_) at “step 1”, followed by excitation of the second VC, which populates the bipartite state |11⟩ (ρ_11,11_) at “step 2”. The population fidelity, $F={\mathrm{Tr}}^{2}\sqrt{\sqrt{\rho_{\mathrm{ideal}}}\rho_{\mathrm{out}}\sqrt{\rho_{\mathrm{ideal}}}}$ , of the CNOT operation is approximately *F* ≈ 0.8 with temperature-dependent dephasing included compared to *F* ≈ 0.91 without. An additional goal of the CNOT operation is to generate entanglement between states |00⟩ and |11⟩, for which its process fidelity is calculated as *F* ≈ 0.41 (see the Supporting Information for details). A maximum coherence (ρ_00,11_) between states |00⟩ and |11⟩ occurs shortly after step 2 (dashed vertical line) in the region where maximum time-specific concurrence (*C* ≈ 0.81) and time-averaged concurrence (*C* ≈ 0.53) are calculated. These values are promising suggesting a high probability of generating coherence and detecting entangled states within the proposed scheme. Remarkedly, as shown in Figure 4, sustained oscillations of the population between states |01⟩ and |11⟩ can be seen on a scale of 10^2^ ps, while excitation of the single-qubit state, |10⟩, is minimized, thus establishing a dynamic repetition of the CNOT operation with increased coherence for the two-photon transition. This time scale, in particular, is more attainable by researchers for the performance of experiments, such as error correction measurements, and for quantum repeaters. The control over populations is demonstrated by manipulating the optical input power and, hence, the plasmonic near field of the NFT, which effectively dictates the energy deposition rate and, thus, nanoscale heating for any initial (cryogenic) temperature. The relationship between the populations (top three rows) and input power is analogous to that of the concurrence (bottom row) given its dependence on occupation and ensuing coherence in the two-photon transition. Hence, an abundance of experimental settings can achieve not only dynamic entanglement of simultaneously emitted photons, but also CNOT operations may be performed via controlled manipulation of the heating, with suitability in probabilistic quantum repeaters or error-correction experiments. We further emphasize the enhancement of the emission rate from the VCs, as witnessed in the Rabi oscillations shown in Figure 4a, with previous works predicting enhancements in similar NFT systems by many orders of magnitude.^11^

**Figure 3:**
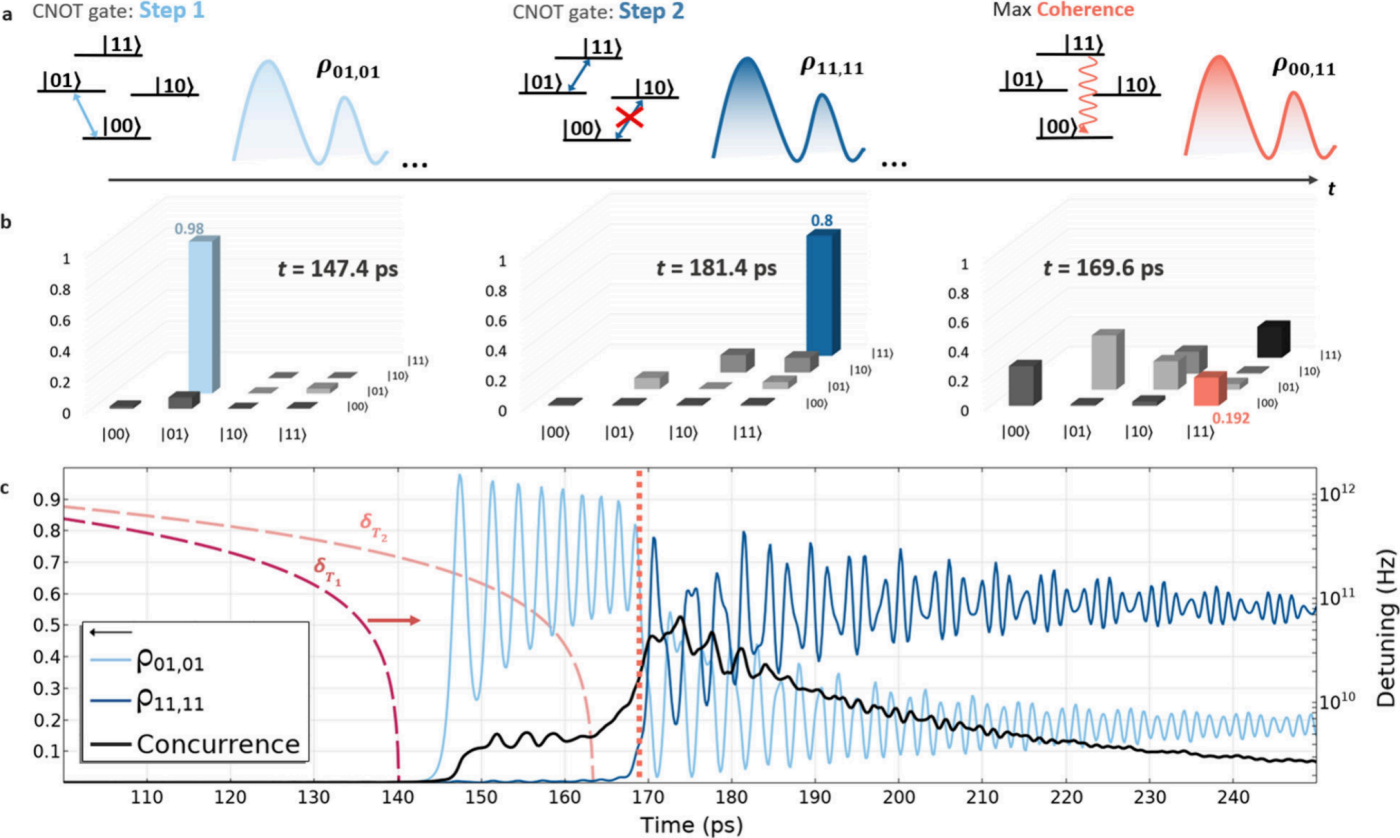
Dynamic CNOT quantum operation using a single excitation pulse. (a) Schematic depicts the time-dependent steps of each quantum operation required on a two-qubit register for a controlled NOT (CNOT) logic gate. Step 1 requires efficient excitation of a single-qubit only, i.e., state |01⟩, with the probability of occupation defined by ρ_01,01_ (×100%), while step 2 ideally excites a bipartite state only, i.e., state |11⟩, defined by ρ_11,11_. Both steps occur as each qubit comes into resonance with the NFT mode when δ_*T*_1__ and δ_*T*_2__ go toward zero (pink/red dashed curves). Each qubit requires a temperature change of roughly 23 K, with *T* ≈ 33 K to be brought into resonance with approximately 22 ps separating the two, i.e., the temporal separation between steps 1 and 2. Maximum probabilities for exciting the desired states are shown in panel b, where a maximum coherence (Re­[ρ_00,11_]) is achieved between |00⟩ and |11⟩ after 169.6 ps (vertical dotted line in panel c) of applied heating and shortly after excitation of the bipartite state when both qubits are tuned into resonance. The population fidelity (*F*) of the CNOT gate is *F* ≈ 0.8 for initialization of the |11⟩ state, whereas the process fidelity is *F* ≈ 0.41 when considering the generation of coherence between states |00⟩ and |11⟩ (see the Supporting Information for details). Panel c demonstrates the dynamics of single-qubit and bipartite state populations for the case of *T*_in_ = 10 K and *P*_in_ = 0.5 mW (time-average concurrence shown). Oscillation between states |01⟩ and |11⟩ while minimizing that of state |10⟩ (shown in Figure 4) is achieved over time scales ranging between 10^1^ and 10^2^ ps, depending on input power and cryogenic temperature. This produces a time-dependent and repetitive CNOT operation with concurrence reaching values above 0.5.

**Figure 4:**
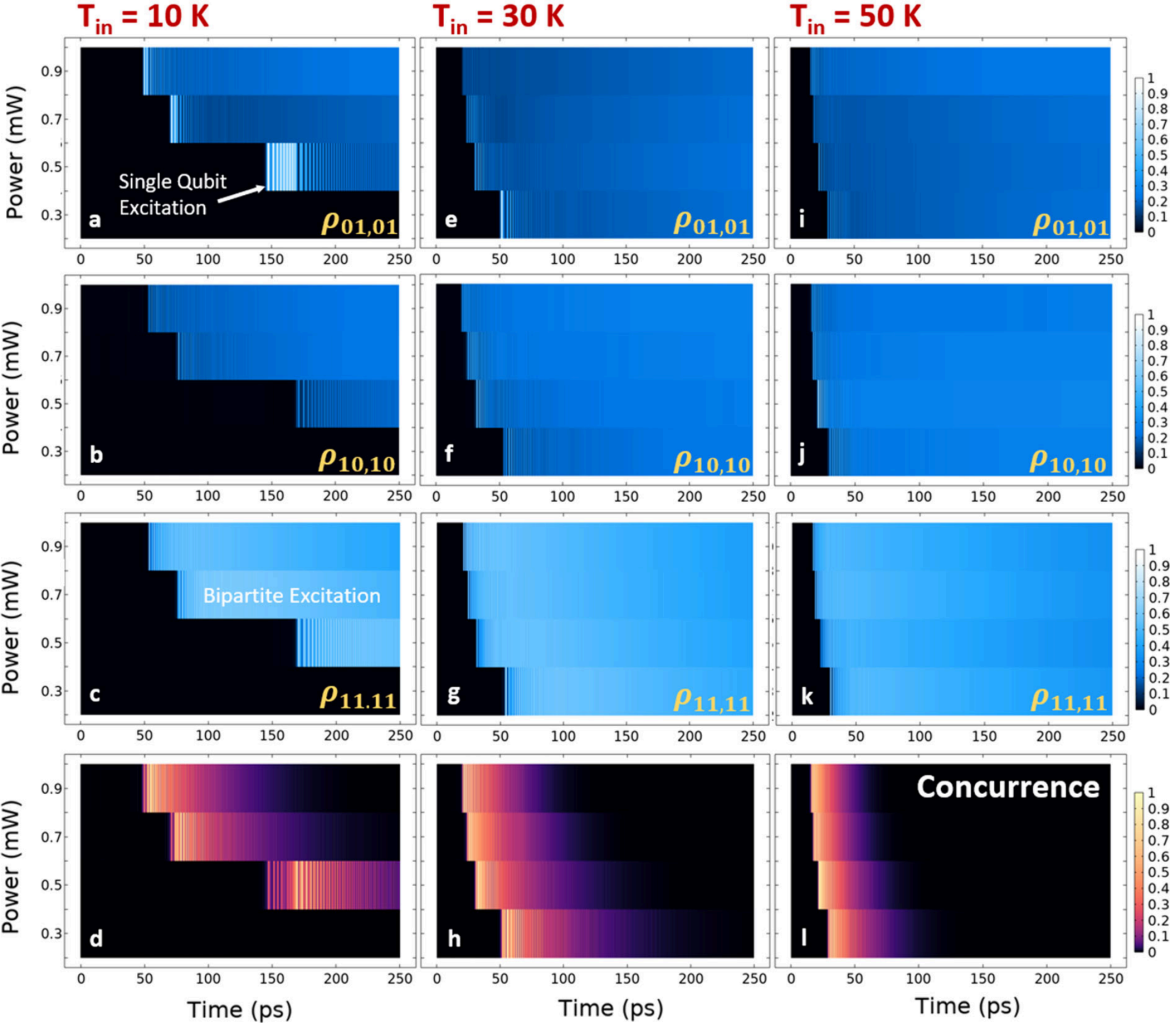
Dynamic excitation of qubit states at elevated temperatures. (a–d) Profiles depict the time dependency of the populations for each state as a function of the input power for *T*_in_ = 10 K. Populations within the two-qubit register are defined from the density matrix with probabilities ρ_01,01_ (×100%) and ρ_10,10_ for individual qubit states, |01⟩ and |10⟩, respectively, and ρ_11,11_ for the bipartite state, |11⟩. Data are taken for input powers of 0.3, 0.5, 0.7, and 0.9 mW with nearest neighbor interpolation used. Panel d is the concurrence as in Figure 2. Panels e–h and i–l are analogous for *T*_in_ = 30 and 50 K, respectively. Dephasing effects clearly attenuate the concurrence, i.e., two-photon coherence, and oscillations between states more rapidly at higher temperatures. Sustained repetition of controlled bipartite excitation is maintained over roughly the same time that concurrence persists, ranging between 10^1^ and 10^2^ ps. Regions of single-qubit excitation, as highlighted in panel a for state |01⟩, may also be exploitable for single-qubit devices. For a period over 50 ps, we see a probability above 50% maintained for the case of bipartite excitation in panel c.

Hitherto, our results have demonstrated dynamic quantum control at temperatures ranging from 10 to 80 K, harnessing silicon VCs embedded in 4H-SiC. For this, the near-field-induced heating and corresponding thermally induced strain enable control of the resonance condition between the emitter’s optical transition and the plasmonic NFT mode. The NFT thus serves a 2-fold purpose: to create the heating, i.e., thermal hot spot, for inducing strain via phonon occupation and to supply the excitation field for the emitters. Furthermore, 4H-SiC is desired given its more substantial absorption coefficient in comparison to a diamond, for example, and therefore its prospects for heat absorption and its manipulation.

To implement the proposed scheme, photon-number detection is required, which may be done by using narrowband spectral filters along with Mach–Zehnder interferometry for retrieving coherence terms. We emphasize that, although we have assumed equal initial ZPL transition energies in this study, indistinguishability, i.e., frequency matching, of the VCs is not a strict requirement and that a frequency mismatch of ZPLs, which may naturally exist, can be accommodated. With the case in Figure 2a (*T*_in_ = 10 K and *P*_in_ = 0.5 mW) highlighted once more, a detuning between ZPL energies of roughly 3.5 THz (≈8.5 nm) still yields a time-averaged concurrence over 0.5 along with a 22 ps timing mismatch when each VC is brought into resonance with the NFT mode. The Debye–Waller (DW) factor (the branching ratio of emission into the ZPL) reflects the level of induced strain in our system, being related to the Huang–Rhys factor, *S*, via DW = e^–*S*^ and, thus, the energy level shift as presented in eq S3 in the Supporting Information. Typical values of 6–9% have been reported for the DW factor of silicon VCs in 4H-SiC; here, we use the more conservative value of 6% in our model, yielding a Huang–Rhys factor of 2.8.^41^ Moreover, we assume an initial detuning of ≈2 THz (4–5 nm) between the targeted ZPL transition of an emitter and the plasmonic NFT mode. Variations in the DW factor and/or the comparative emitter ZPL energies, for instance, due to inhomogeneous strain, relative to our studied configuration, could be accommodated via modulation of the optical energy deposition [dependent on the NFT position/input power (see the Supporting Information for a detailed example)] or through the application of an initial mechanical strain field to adjust the transition energies appropriately.

We conclude by emphasizing the viability of our scheme for achieving quantum operations at elevated temperatures, where optically emitting defect centers, i.e., color centers, with appropriately long dephasing times are brought into resonance with a nanoplasmonic resonator mode using controlled, near-field-induced heating. At temperatures of a few tens of kelvin, our simulations suggest the capability to create entangled photon-number states given appropriate filtering as well as sustained population control and coherence that is time-repetitive over scales of 10^1^–10^2^ ps. Moreover, the adaptiveness of the NFT, as the source of the plasmonic near field, along with using Si or other group IV color centers as qubits enables promising opportunities toward scalable manufacturing, e.g., for spin-stand compatibility, multipartite entanglement applications, as well as tunable quantum photonic technologies with improved fidelities of entanglement.^15,42^

## Supplementary Material



## Notes

Theoretical methods, density matrix equations, derivation of concurrence, quantification of fidelity calculations, full parameter list, workflow schematic, additional figure on varying dipole moment strengths, schematic of the near-field transducer, images of photonic and plasmonic waveguide mode, and additional figure on the effect of NFT rastering on concurrence and emitter temperatures (PDF)

## ‡

Institute of Physics, University of Münster, Wilhelm-Klemm-Straße 10, 48149 Münster, Germany

## Abbreviations Used

VC: vacancy center
NFT: near-field transducer
*V*_Si_^–^: silicon vacancy
4H-SiC: 4H polytype silicon carbide
ZPL: zero-phonon line
CNOT: controlled NOT
DW: Debye–Waller
