# Supplementary material for: Dynamic Quantum Operations at Elevated Temperatures Using Hot-Spot Nanoheating of Color Centers
Source: Nano Lett. 2025 Aug 28;25(37):13935–42. doi: 10.1021/acs.nanolett.5c04008 (PMC12447548; doi:10.1021/acs.nanolett.5c04008)
Supplement: Supplementary file 1 [file nl5c04008_si_001.pdf]

Supplementary Text For:

Dynamic Quantum Operations at Elevated Temperatures using Hot-Spot Nanoheating of Color Centers

Frank D. Bello[1], Daniel D. A. Clarke[1], Daniel Wigger[1], and Ortwin Hess[1,2*]*

[1]School of Physics and CRANN, Trinity College Dublin, Dublin 2, Ireland

[2]Advanced Materials and Bioengineering Research (AMBER) Center, Trinity College Dublin, Dublin 2, Ireland

*Corresponding authors: fbello@tcd.ie, ortwin.hess@tcd.ie

Theoretical Methods

We use finite-element time domain (FETD) simulations to compute the electric field and temperature distributions that are experienced by the V_{Si}^{-} centers in 4H-SiC[1]. The full-field, steady state solution of the Helmholtz equation and thermal diffusion equation is solved for a photonic waveguide that is coupled to a metal-insulator-semiconductor (MIS) plasmonic resonator which is placed above a media stack. The photonic waveguide is designed for single mode operation at a wavelength of 866 nm, though this may be adjusted to accommodate a wide range of ZPL energy shifts as a function of temperature or material used[4]. Previously, we have shown that dipole moments are able to strongly interact with any electric field component emitted from the MIS resonator, i.e., the NFT, whose values range between 10^6-10^8 V/m[5]. The media is composed of Air/lubricant (2.5 nm thick), Silicon Carbide (30 nm), and a Silicon Dioxide substrate (100 nm). Please see Tables 1 and 2 of the Supplementary Information for a full list of dimensions and a schematic of the media[2].

Semiclassical Approach to Electrodynamics

We separately calculate for the time-dependent density matrix of the two coupled emitters forming the 4-level system, and remove the waveguide core and MIS resonator from numerical simulations, considering the incident electric field and temperature distribution as their primary contribution to the time dynamics of the Si-vacancy layer/media. This method has been successfully used to model the power dissipation and heat transfer dynamics in similar NFT + media systems[6]. An average of the electric field[7] and temperature within the region outlined for the vacancy center is used in the numerics.

The effect of the V_{Si}^{-} centers on their electromagnetic environment is included within Maxwell's (Helmholtz) equations by computing the polarization arising from their dipole moment,

$$\varepsilon \partial_t \mathbf{E} = \nabla \times \mathbf{H} - \partial_t \mathbf{P}, \quad \text{(S1)}$$

where all variables are functions of time and 3-dimensional space. **P**, defined below, is calculated at the vacancy's position ($\boldsymbol{r}$) which requires computing the ensemble averaged polarization (**p**) for each VC using the appropriate density matrix ($\hat{\rho}$) element,

$$\mathbf{P} = \langle \mathbf{p} \rangle = \eta \mathrm{Tr}(\hat{\mu}\hat{\rho}) \quad \text{(S2)}$$

Here, the dipole moment ($\hat{\mu}$) is assumed to be real and equal for each VC, with $\mu_{ij} = \langle i|\hat{\mu}|j\rangle = \mu_{ji}$, and $\eta \approx 1/\mathrm{nm}^3$ is taken as the density of each color center. To calculate the density matrix (See Supplementary Information for details), we first define the Hamiltonian using raising and lowering operators, $\sigma_i^{\pm}$, for a 2-qubit (4×4 matrix, i=1,2) system,

$$H = \sum_{i=1,2} \hbar\omega_i \sigma_i^{+}\sigma_i - 2S\hbar\omega_{ph}\langle n_{ph}(T)\rangle \sigma_i^{+}\sigma_i + \hat{\mu} \cdot \mathrm{E}_i^{sc}(\sigma_i^{+} + \sigma_i). \quad \text{(S3)}$$

Here, ω_i is the frequency of the zero-phonon line, often designated as V1 and found near 861.5 nm for V_{Si}^{-} centers located at hexagonal sites in 4H-SiC. It is at this wavelength where the emission of entangled pairs of photons is anticipated.

We consider color centers which are aligned along the c-axis of the SiC crystal and treat the effect of phonon occupation via a temperature-dependent shift to the transition energies. In other words, the phonon occupation is anticipated to affect purely the diagonal elements of the Hamiltonian and thus the ZPL positions. The change to the transition energies is defined by the second term of Eq. (3), where S is the material dependent Huang-Rhys factor and $\langle n_{ph} \rangle = [\exp(\frac{\hbar\omega_{ph}}{k_B T} - 1)]^{-1}$ is the average phonon number with corresponding frequency $\omega_{ph} = 17$ THz. Of note, a coherent coupling term between two quantum emitters is not explicit in the Hamiltonian, rather it is implicitly incorporated via the spatial integration of the electromagnetic field, which includes the polarization of Eq. (S2), throughout the media.

Heating Dynamics

The temperature throughout the media is calculated from the time-dependent thermal diffusion equation,

$$\partial_t T = D_T \nabla^2 T + Q_{Res} \quad \text{(S4)}$$

D_T is the thermal diffusion coefficient, while the heat source is taken to be the electromagnetic power loss in the structure, i.e. resistive or Joule heating (Q_{Res}). The majority of heat produced in the 4H-SiC is anticipated to occur within 100 ps as the estimated electron heating (femtosecond scale) and ensuing lattice heating (picosecond scale) move the system towards steady state temperature values[8]. At temperatures below 50 K, single-acoustic-phonon scattering processes are expected to be the primary source of pure dephasing, with a temperature-dependent rate given by $\gamma_{Tij}(T) = A{\Delta_a}^3[\exp(\frac{\hbar\Delta_a}{k_B T} - 1)]^{-1}$ [9]. Here Δ_a is the polaronic gap of the V1 center in 4H-SiC, approximately 4.3 meV, while A is an empirically derived fitting parameter found to be 0.34 GHz/meV^3 [10]. Above 50 K a cubic temperature dependence on dephasing is adopted based on silicon VCs in diamond, defined as $\gamma_{Tij}(T) = (103 + 0.12[T/\mathrm{K}]^3)\mathrm{MHz}$ [11]. The values for the exponential and cubic temperature-dependent dephasing are approximately equal at 50 K.

Density Matrix

The quantum master equation for the time-dependent density matrix is given by

$$i\hbar\partial_t\hat{\rho} = [\hat{H}, \hat{\rho}] - i\hbar\hat{\Gamma}(\hat{\rho}) \tag{S5}$$

where $\hat{\Gamma}$ is a super operator that includes the radiative decay (γ_r) and temperature-dependent dephasing rates ($\gamma_T(T)$) of each energy level with $\hat{\Gamma}(\hat{\rho})$ for each vacancy center defined as

$$\hat{\Gamma}(\hat{\rho}) = \frac{\gamma_r}{2}(2\sigma_1\hat{\rho}\sigma_1^+ - \sigma_1^+\sigma_1\hat{\rho} - \hat{\rho}\sigma_1^+\sigma_1) + \frac{\gamma_{T01}(T)}{2}(2\sigma_{z1}\hat{\rho}\sigma_{z1}^+ - \hat{\rho}) + \frac{\gamma_r}{2}(2\sigma_2\hat{\rho}\sigma_2^+ - \sigma_2^+\sigma_2\hat{\rho} - \hat{\rho}\sigma_2^+\sigma_2) + \frac{\gamma_{T10}(T)}{2}(2\sigma_{z2}\hat{\rho}\sigma_{z2}^+ - \hat{\rho}). \tag{S6}$$

We use a symmetry-adapted averaging approach of the electric field to exclude all self-interactions the quantum emitter may have with its emitted field(*35*). Therefore, the scattered field used in the Hamiltonian is defined as an average of the full field solution in the region of the vacancy center,

$$\mathrm{E}_i^{\mathrm{sc}} = \frac{1}{N}\sum_j \mathbf{E}(\mathrm{r}_j), \tag{S7}$$

where '*N*' is the number of data points defined within the vacancy with the summation going from 1 to *N*. A flow chart is depicted in Figure S1 showing the order of calculations taken in the simulation.

Each vacancy center is modeled as a 2-level quantum system with respect to the ZPL transition, where a general single-qubit state is given by $|\Psi\rangle = \alpha|0\rangle + \beta|1\rangle$ and $\alpha\alpha^*$ and $\beta\beta^*$ are the probabilities to find the emitter in either the ground or excited state, respectively. For a two-qubit system a general state is formed by taking the tensor product $|\varphi\rangle = |\Psi_1\rangle \otimes |\Psi_2\rangle$, yielding $|\varphi\rangle = \alpha_1\alpha_2|00\rangle + \alpha_1\beta_2|01\rangle + \beta_1\alpha_2|10\rangle + \beta_1\beta_2|11\rangle$. $\hat{\rho}$ of Eq. S1 is a function of the wave function used, for example $\hat{\rho} = |\varphi\rangle\langle\varphi|$ for the bipartite system. Subscripts used for the matrix elements have i or j labels using 00, 01, 10, or 11 for each, which corresponds to the 4 bipartite basis states. The density matrix elements below are derived by applying the appropriate bra ('i' basis state) and ket ('j' basis state) to Eq. S1. The diagonal elements yield the populations of each level while the off-diagonal elements are the coherence terms that are necessary to calculate the polarization in

Eq. 2 of the main text. Please see Fig. 1A for a schematic of the 4-level system utilized to describe the bipartite configuration.

We solve the following density matrix equations (dipole aligned in z-direction) while ensuring the trace $\sum \rho_{ii} = 1$ is conserved. Within finite-element simulations, a minimum element size of 2 nm is used with a maximum error of 0.001 allowed. The incident electric field profile is considered to have a monochromatic time dependence ($e^{-i\omega_L t}$), while the detunings, δ_{T_i}, between each shifted energy level and the laser (ω_L) are given by

$$\delta_{T_i} = \hbar\omega_i - \hbar\omega_L - 2S\hbar\omega_{ph}\langle n_{ph}\rangle \tag{S8}$$

<u>Quantifying the Two-Photon Filter/Interferometry</u>

Given the density matrix of the two-qubit system, $\hat{\rho}$, a suitable experimental filter, $\hat{F}$, is envisaged post-emission to distill photon pairs, for example, using narrowband spectral filters, along with tomography of the photon-number entangled states[12, 13], $|00\rangle$ and $|11\rangle$, Capturing such an extrinsic filtering operation via the matrix

$$\hat{G} = \begin{pmatrix} 1 & 0 & 0 & 0 \\ 0 & 0 & 0 & 0 \\ 0 & 0 & 0 & 0 \\ 0 & 0 & 0 & e^{i\varphi} \end{pmatrix}, \tag{S9}$$

the transformation $\hat{G}\hat{\rho}\hat{G}^{\dagger}/\mathrm{Tr}(\hat{G}\hat{\rho}\hat{G}^{\dagger})$ yields a two-photon density matrix,

$$\hat{\rho}^{2p} = \begin{pmatrix} \rho_{00,00} & 0 & 0 & \rho_{00,11}e^{-i\varphi} \\ 0 & 0 & 0 & 0 \\ 0 & 0 & 0 & 0 \\ \rho_{11,00}e^{i\varphi} & 0 & 0 & \rho_{11.11} \end{pmatrix} \times \frac{1}{\rho_{00,00}+\rho_{11,11}} \tag{S10}$$

where the denominator is a normalization factor and φ is the relative phase introduced using Mach-Zehnder interferometry in order to perform state tomography and retrieve coherence terms[14]. We then evaluate the concurrence of the reduced density matrix by calculating the eigenvalues of $\hat{\rho}^{2p}(\hat{\sigma}_y \otimes \hat{\sigma}_y)\hat{\rho}^{2p*}(\hat{\sigma}_y \otimes \hat{\sigma}_y)$, where $\hat{\sigma}_y$ is a Pauli spin matrix[15]. The concurrence is defined as $C = \max(0, \sqrt{\lambda_1} - \sqrt{\lambda_2})$, where the eigenvalues, $\lambda_{1,2}$, are numbered in decreasing order. The two eigenvalues are found to be $\lambda_{1,2} = (\sqrt{\rho_{00,00}\rho_{11,11}} \pm |\rho_{00,11}|)^2$, which yields $C = 2|\rho_{00,11}|/(\rho_{00,00} + \rho_{11,11})$ when including the normalization factor[16].

We note that values are time-dependent and, hence, a time-averaged result is used to describe the measured concurrence, considering additional sources of dephasing post-emission. For instance, deviations from ideal spectral filtering and photodetection are expected to further reduce the concurrence values from those simulated. Specifically, we define the time-averaged concurrence via

$$C(t) = \frac{1}{t}\int_0^t d\tau\, 2|\rho_{00,11}^N(\tau)|. \qquad \text{(S11)}$$

.

Fig. S1 has a flow chart that depicts the computational methods used for each time step and how parameters are fed into the equations.

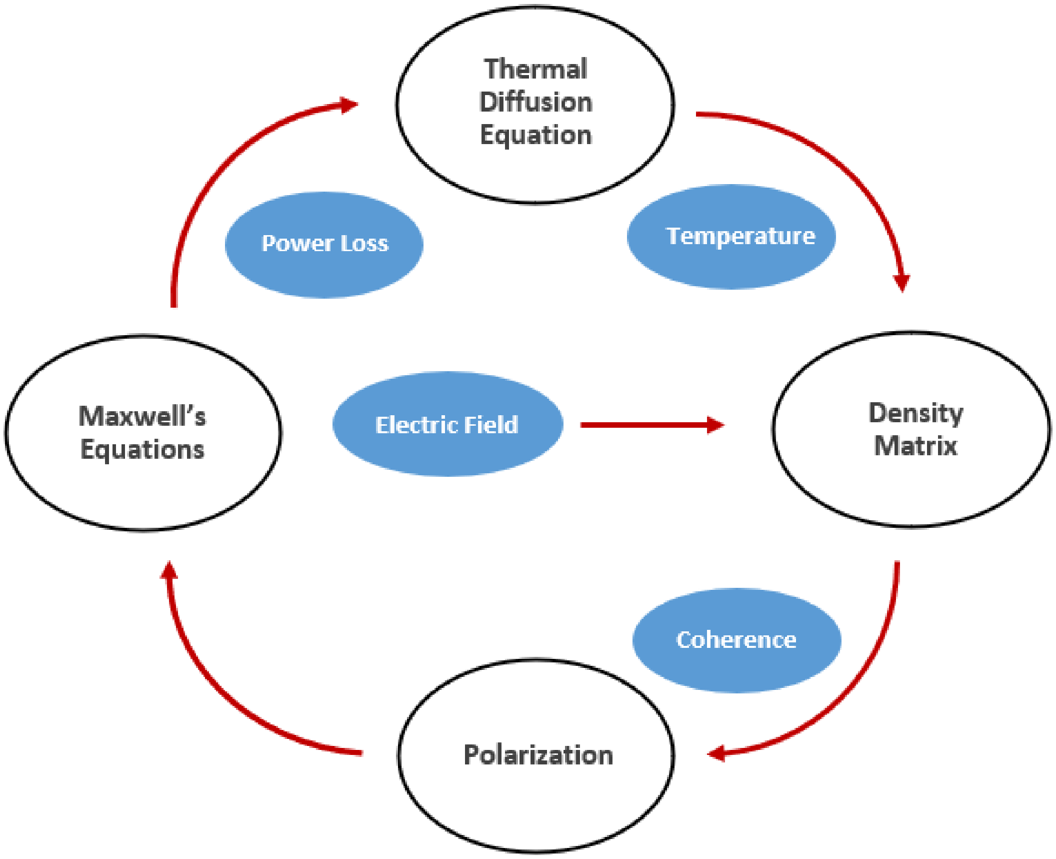

Fig. S1. Flow chart for time-dependent simulations and parameters. A flow chart showing the calculations taking place in the time-dependent simulation. The Helmholtz equation is solved first, with solutions for the electric field being supplied to the density matrix simulation and the power loss (resistive heat source) to the thermal diffusion equation for each time step. Simultaneously the density matrix is solved where the coherence (from the off-diagonal elements) for each vacancy center is used to calculate the polarization. The polarization is fed back into the Helmholtz equation and the process is repeated for each time step.

List of Density Matrix Equations

$$\dot{\rho}_{00,00} = \frac{i}{\hbar}\mu\mathrm{E}_z(r_2)\big(\rho_{00.01} - \rho_{01,00}\big) + \frac{i}{\hbar}\mu\mathrm{E}_z(r_1)\big(\rho_{00,10} - \rho_{10,00}\big) + \gamma_{12}\rho_{01,01} + \gamma_{13}\rho_{10,10}$$

$$\dot{\rho}_{01,01} = -\frac{i}{\hbar}\mu\mathrm{E}_z(r_2)\big(\rho_{00,01} - \rho_{01,00}\big) + \frac{i}{\hbar}\mu\mathrm{E}_z(r_1)\big(\rho_{01,11} - \rho_{11.01}\big) + \gamma_{13}\rho_{11,11} - \gamma_{12}\rho_{01,01}$$

$$\dot{\rho}_{10,10} = \frac{i}{\hbar}\mu\mathrm{E}_z(r_2)\big(\rho_{10,11} - \rho_{11,10}\big) - \frac{i}{\hbar}\mu\mathrm{E}_z(r_1)\big(\rho_{00,10} - \rho_{10,00}\big) + \gamma_{12}\rho_{11,11} - \gamma_{13}\rho_{10,10}$$

$$\dot{\rho}_{11,11} = -\frac{i}{\hbar}\mu\mathrm{E}_z(r_2)\big(\rho_{10,11} - \rho_{11,10}\big) - \frac{i}{\hbar}\mu\mathrm{E}_z(r_1)\big(\rho_{01,11} - \rho_{11,01}\big) - \gamma_{12}\rho_{11,11} - \gamma_{13}\rho_{11,11}$$

$$\dot{\rho}_{00,10} = i\delta_{T_2}\rho_{00,01} - \frac{i}{\hbar}\mu\mathrm{E}_z(r_2)\big(\rho_{00,00} - \rho_{01,01}\big) - \frac{i}{\hbar}\mu\mathrm{E}_z(r_1)\big(\rho_{00,11} - \rho_{10,01}\big) - (\frac{\gamma_{12}}{2} + \gamma_{T12}(T))\rho_{00,01} + \gamma_{13}\rho_{10,11}$$

$$\dot{\rho}_{00,10} = i\delta_{T_1}\rho_{00,10} + \frac{i}{\hbar}\mu\mathrm{E}_z(r_2)\big(\rho_{00,11} - \rho_{01,10}\big) + \frac{i}{\hbar}\mu\mathrm{E}_z(r_1)\big(\rho_{00,00} - \rho_{10,10}\big) - (\frac{\gamma_{13}}{2} + \gamma_{T13}(T))\rho_{00,10} + \gamma_{12}\rho_{01,11}$$

$$\dot{\rho}_{01,11} = i\delta_{T_1}\rho_{01,11} - \frac{i}{\hbar}\mu\mathrm{E}_z(r_2)\big(\rho_{00,11} - \rho_{01,10}\big) + \frac{i}{\hbar}\mu\mathrm{E}_z(r_1)\big(\rho_{01,01} - \rho_{11,11}\big) - (\frac{\gamma_{13}}{2} + \gamma_{T13}(T) + \gamma_{12})\rho_{01,11}$$

$$\dot{\rho}_{10,11} = i\delta_{T_2}\rho_{10,11} + \frac{i}{\hbar}\mu\mathrm{E}_z(r_2)\big(\rho_{10,10} - \rho_{11,11}\big) - \frac{i}{\hbar}\mu\mathrm{E}_z(r_1)\big(\rho_{00,11} - \rho_{10,01}\big) - (\frac{\gamma_{12}}{2} + \gamma_{T12}(T) + \gamma_{13})\rho_{10,11}$$

$$\dot{\rho}_{00,11} = i(\delta_{T_1} + \delta_{T_2})\rho_{00,11} + \frac{i}{\hbar}\mu\mathrm{E}_z(r_2)\big(\rho_{00,10} - \rho_{01,11}\big) + \frac{i}{\hbar}\mu\mathrm{E}_z(r_1)\big(\rho_{00,01} - \rho_{10,11}\big) - (\gamma_{T12}(T) + \gamma_{T13}(T))\rho_{00,11} - \frac{(\gamma_{12} + \gamma_{13})}{2}\rho_{00,11}$$

$$\dot{\rho}_{01,10} = i(\delta_{T_1} - \delta_{T_2})\rho_{01,10} + \frac{i}{\hbar}\mu\mathrm{E}_z(r_2)\big(\rho_{01,11} - \rho_{00,10}\big) + \frac{i}{\hbar}\mu\mathrm{E}_z(r_1)\big(\rho_{01,00} - \rho_{11,10}\big) - (\gamma_{T12}(T) + \gamma_{T13}(T))\rho_{01,10} - \frac{(\gamma_{12} + \gamma_{13})}{2}\rho_{01,10}$$

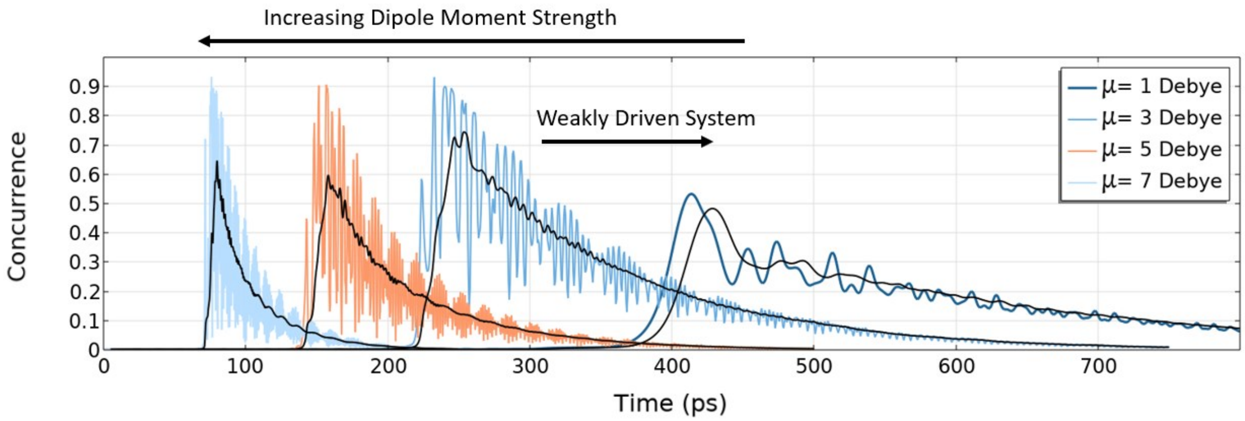

Fig. S2. Concurrence as a function of varying dipole moments. Concurrence is shown for varying strengths of the dipole moment given the case of T_{in}= 10 K and P_{in}= 0.7 mW (orange curve of Figure 2a) with time-averaged concurrence (every 5 ps) shown in overlapping black curves. The time-average is begun at 5 ps into the simulation. Concurrence for each scenario begins at the same time as the far-left curve though curves are offset from each other to make viewing easier. Results show a noticeable reduction in concurrence as we move towards a weakly driven system for a dipole moment strength of 1 Debye. Maximum concurrence is comparable for larger values of the dipole moment within a strongly driven system, however it attenuates towards 0 faster for larger values. As the dipole moment increases so do Rabi oscillation rates of individual qubits, which in turn slightly reduces coherence between the bipartite and ground states and therefore the concurrence of simultaneously-emitted entangled pairs of photons.

Table S1: Photonic and Plasmonic Waveguide Dimensions[2]

**Waveguide Dimensions, (Material, refractive index)**	
Si waveguide - length (z), width (x)	0.5 µm, 450 nm
Si waveguide - thickness (y)	255 nm
Au film - thickness	60 nm
Taper /Au/SiO_2/Si - length	330 nm
Au, SiO_2, Si tip - width	20 nm
SiO_2 - thickness	10 nm
Metal Cladding (Fe/FeOxide, not shown) - length, width	50 nm, 520 nm
Metal Cladding - thickness	100 nm
Au absorber - length, width	222 nm, 450 nm
Au absorber - thickness	250 nm
**Media Dimensions**	
Air/Lube (x, y, z)	0.9 µm, 1.05 µm , 2.5 nm
SiC (SiV layer)	0.9 µm, 1.05 µm , 30 nm
Si Substrate	0.9 µm, 1.05 µm , 100 nm

Table S2: Thermal Parameters and Refractive Indices[2]

**Film (thickness)**	**Heat Capacity ($J\cdot m^{-3}K^{-1}$)**	**Thermal Conductivity ($W\cdot m^{-1}K^{-1}$)**	**Refractive Index (830 nm)**
Heated Air/Lubricant (2.5 nm)	1.0×10^{6}	3.0	2.9+0.12i
SiC (30 nm)	2.384×10^{6}	1.44	3.3067+0.14389i
SiO_2 Substrate (100 nm)	1.606×10^{6}	1.4	1.4528
**Materials (NFT, waveguide)**			
Gold	2.4897×10^{6}	317	0.19988+4.9374i
SiO_2	1.606×10^{6}	1.4	1.4528
Si	1.6303×10^{6}	130	3.673
Metal cladding	3.73×10^{6}	3.0	2.1415+4.2361i

Table S3: Parameters used in Density Matrix Simulations

**Vacancy Center Parameters**	
Radiative decay rates	
$\gamma_{12}, \gamma_{13} = \gamma_{ij}$	1 GHz
Dipole moment	
μ (same for both emitters)	5 Debye (Reports up to 14 D for Si VCs[17])
Huang-Rhys factor *(S)*	2.8[18]
Average phonon energy (ω_{ph})	17 THz[19]

Quantifying Fidelity

Uhlmann-Jozsa Fidelity (F) quantifies the overlap of the ideal output state (ρ_{ideal}) of a quantum operation in relation to the actual output state (ρ_{out}) and is given by,

$$F = \left(\mathrm{Tr}\sqrt{\sqrt{\rho_{\text{ideal}}}\rho_{\text{out}}\sqrt{\rho_{\text{ideal}}}}\right)^2,$$

where ρ is the density matrix. We report on the fidelity for the purpose of initializing a computational basis state, i.e. state or population fidelity, which is a common logic operation for classical or quantum computing. Here, the ideal state achieved by the CNOT operation is $|11\rangle$ via excitation of a single-qubit state with the CNOT operation defined as

$$\text{CNOT} = \begin{pmatrix} 1 & 0 & 0 & 0 \\ 0 & 1 & 0 & 0 \\ 0 & 0 & 0 & 1 \\ 0 & 0 & 1 & 0 \end{pmatrix}.$$

Assuming successful initialization i.e.

$$\rho_{\text{initial}} = \begin{pmatrix} 0 & 0 & 0 & 0 \\ 0 & 0 & 0 & 0 \\ 0 & 0 & 1 & 0 \\ 0 & 0 & 0 & 0 \end{pmatrix},$$

this yields a fidelity given by ρ_{44} of the actual ouput matrix with

$$\rho_{\text{ideal}} = \text{CNOT}.\, \rho_{\text{initial}}.\, \text{CNOT}^{\dagger} = \begin{pmatrix} 0 & 0 & 0 & 0 \\ 0 & 0 & 0 & 0 \\ 0 & 0 & 0 & 0 \\ 0 & 0 & 0 & 1 \end{pmatrix}.$$

Fidelities are then $F = 0.81$ for the case presented in Figure 3 of the main text, which increases to $F = 0.91$ when temperature-dependent dephasing is neglected.

Alternatively, the process fidelity measures the accuracy of steps taken by the CNOT operation to achieve the population of state $|11\rangle$, which should generate quantum coherence and entanglement such that the Bell State $(|00\rangle + |11\rangle)/\sqrt{2}$ is formed. We then have the ideal outcome formulated as,

$$\rho_{\text{ideal}} = \frac{1}{2}\begin{pmatrix} 1 & 0 & 0 & 1 \\ 0 & 0 & 0 & 0 \\ 0 & 0 & 0 & 0 \\ 1 & 0 & 0 & 1 \end{pmatrix}.$$

This yields a 'process' fidelity of $F = 0.41$ when taking ρ_{out} at the time of maximum population of state $|11\rangle$. This increases to $F = 0.434$ when neglecting temperature-dependent dephasing. Though relatively low, we emphasize the logic gate fulfills the general purpose of generating coherence, from which entanglement (measured as concurrence) is then improveed by the proposition of photon-number filtering, post emission.

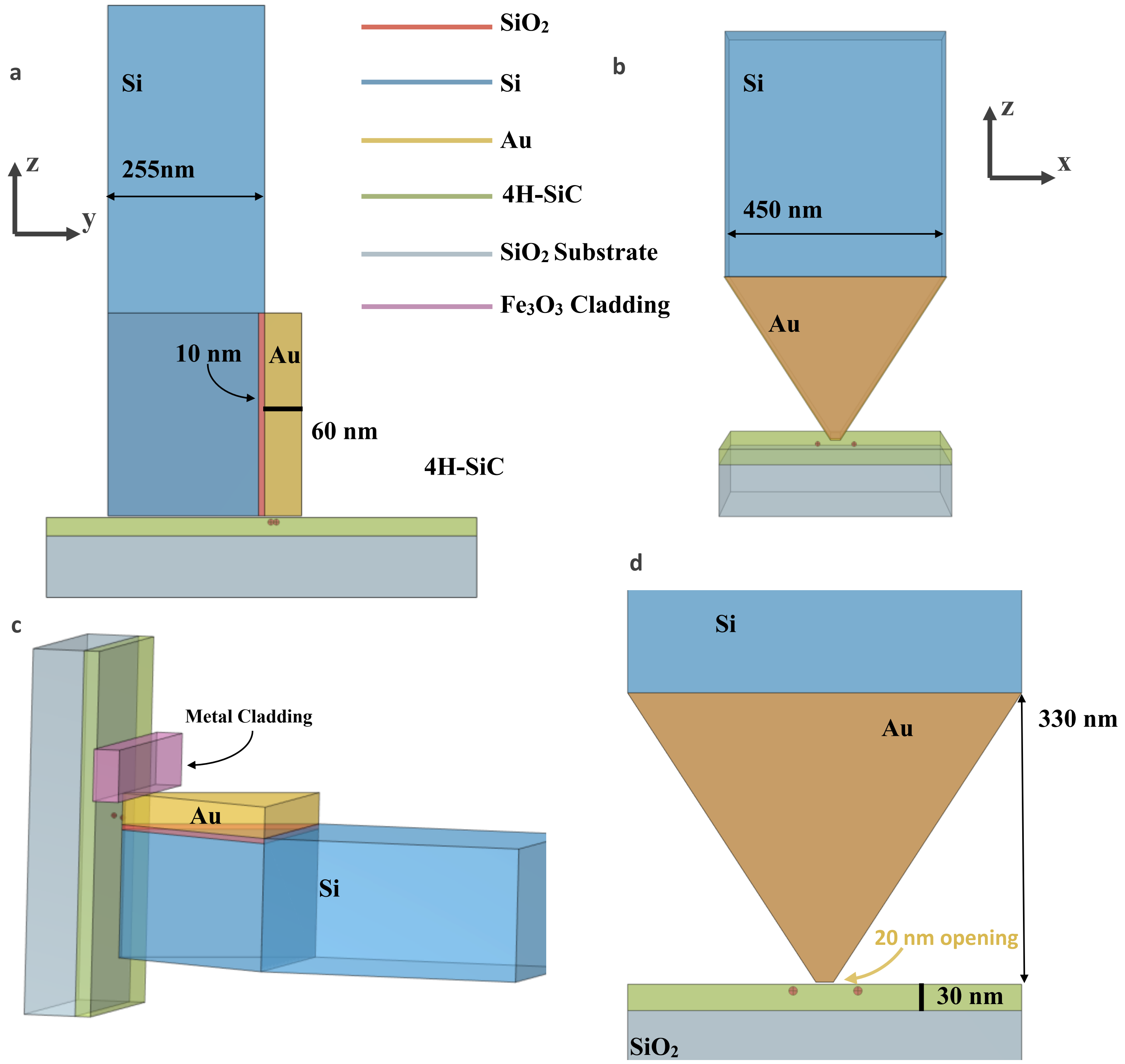

Fig S3. To-scale model of the metal-insulator-semiconductor (MIS), antenna-based plasmonic waveguide which comprises the NFT designed to be fabricated with layer deposition and etching (no e-beam lithography required[2, 3]). Depending on the density of color centers and desired maximum temperature, a metal cladding, though not required, was included in the simulation that can further control the nanoscale heating region of the 'hot spot' and alter thermal gradients a few K/nm. Sample dimensions are displayed with complete dimensions in Table S1. Within the simulation the entire photonic waveguide + NFT structure is cladded in SiO_2. Of note, an additional Au absorber could be also added to reduce unwanted reflections and scattering to/from the media, however we remark that it has no discernible effect on the hot spot size for this structure (See Fig. S4).

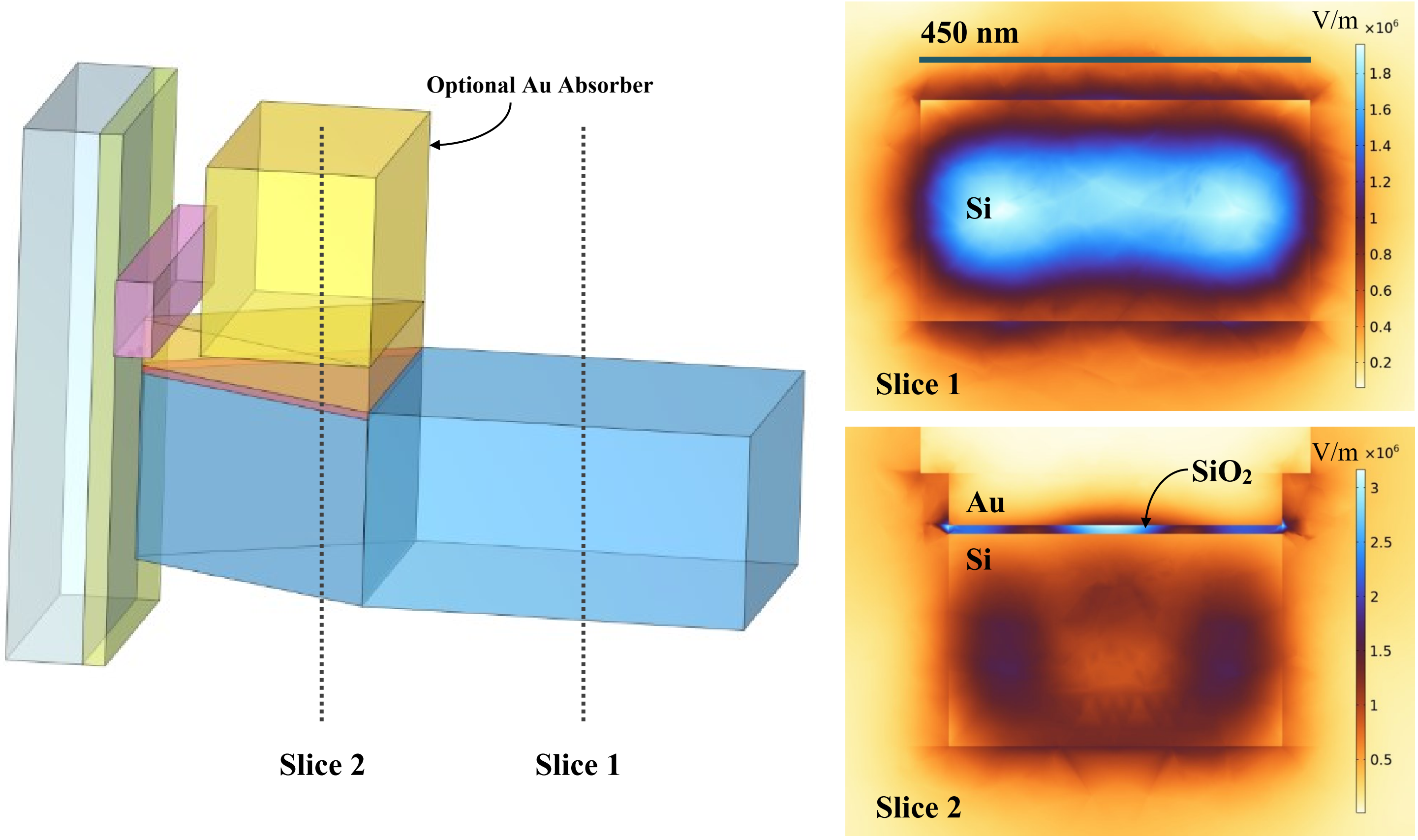

Fig S4. To-scale model (left) with an optional Au absorber added to the back end of the taper to prevent unwanted reflections or scattering of light, primarily from the top of the NFT or photonic WG, particularly if the Au taper is desired to be reduced in thickness. It has no discernible effect on hot spot size shown in Fig. 1 of the main text. Using an input power of 0.5 mW (corresponding to Fig. 1 of the main text), **Slice 1** shows the single-mode TM photonic waveguide at the center of the Si slab. **Slice 2**, taken 50 nm from the beginning of the taper, demonstrates excitation of the plasmonic mode with largest electric field values lying within the SiO_2 layer. Both slices are the same size.

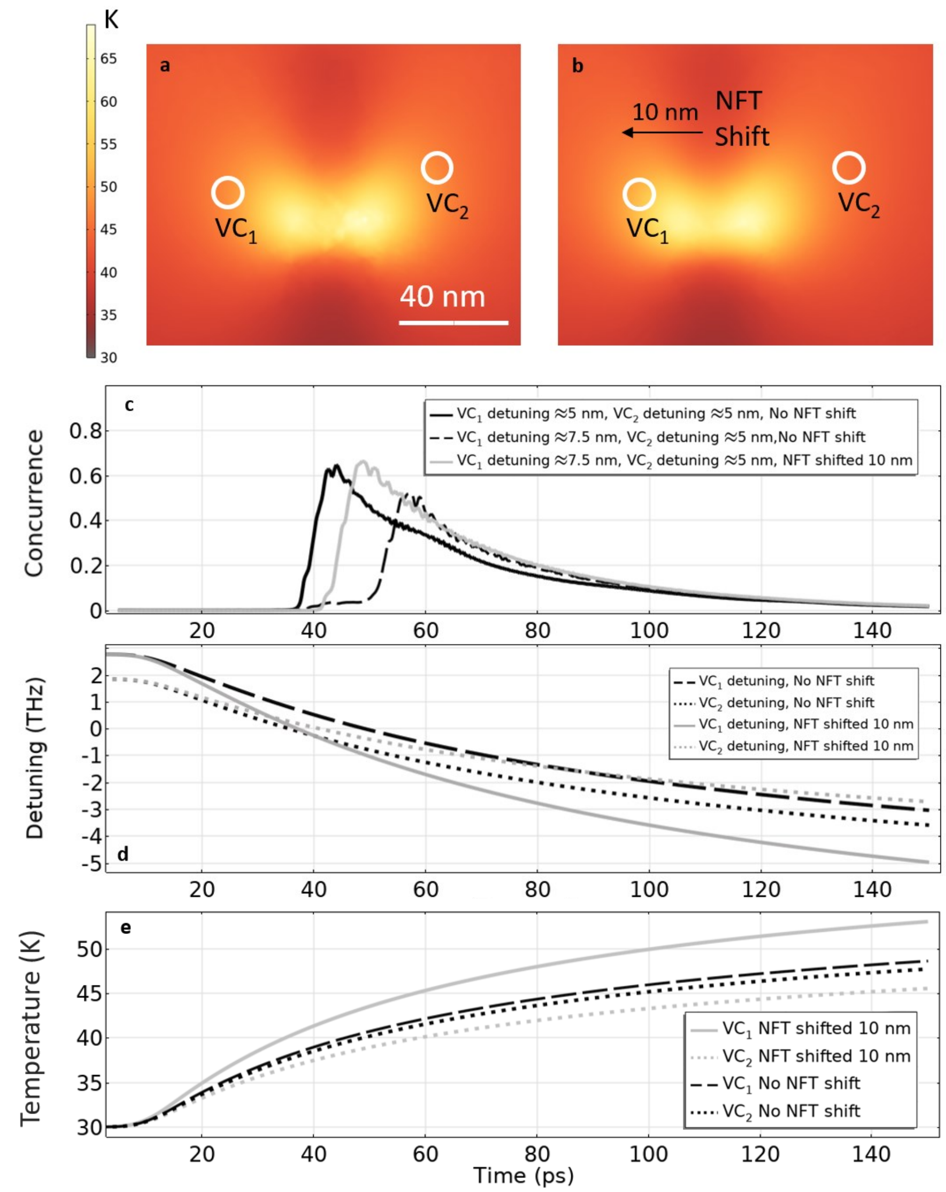

Fig S5. (a) Temperature is shown after 150 ps for an input power of 0.4 mW with an initial cryogenic temperature of 30 K, which yields a maximum time-averaged concurrence value of approximately 0.65 (solid black curve in **(c)**). If the initial detuning of one of the color centers (VC_1) is increased to 7.5 nm (×1.5), we notice the maximum time-averaged concurrence is reduced to roughly 0.5 (dashed curve in **(c)**). In **(b)** we demonstrate by moving the NFT 10 nm to the left that the VCs now experience different maximum temperatures, and thus by changing the heating rate of each VC, we therefore can change the rate of tuning. This allows the ZPL energies of each VC to be closer in resonance with each other circa the time they become on resonant with the plasmonic near field, as shown by the solid and dotted gray curves in Fig. **(d)**. The concurrence once again achieves values greater than 0.65 (gray curve in **(c)**). The increased detuning of VC_1 with no shift of the NFT keeps the VCs away from resonance with each other while also increasing the timing mismatch when they are on resonant with the near-field, as shown by the dashed and dotted black curves in **(d)**. Hence, rastering of the NFT's position is a convenient tool to for adjusting the heating rates of each VC as shown if Fig. (e). The faster and higher change in temperature at the location of VC1 (gray solid curve) is able to compensate for the detuning mismatch created.

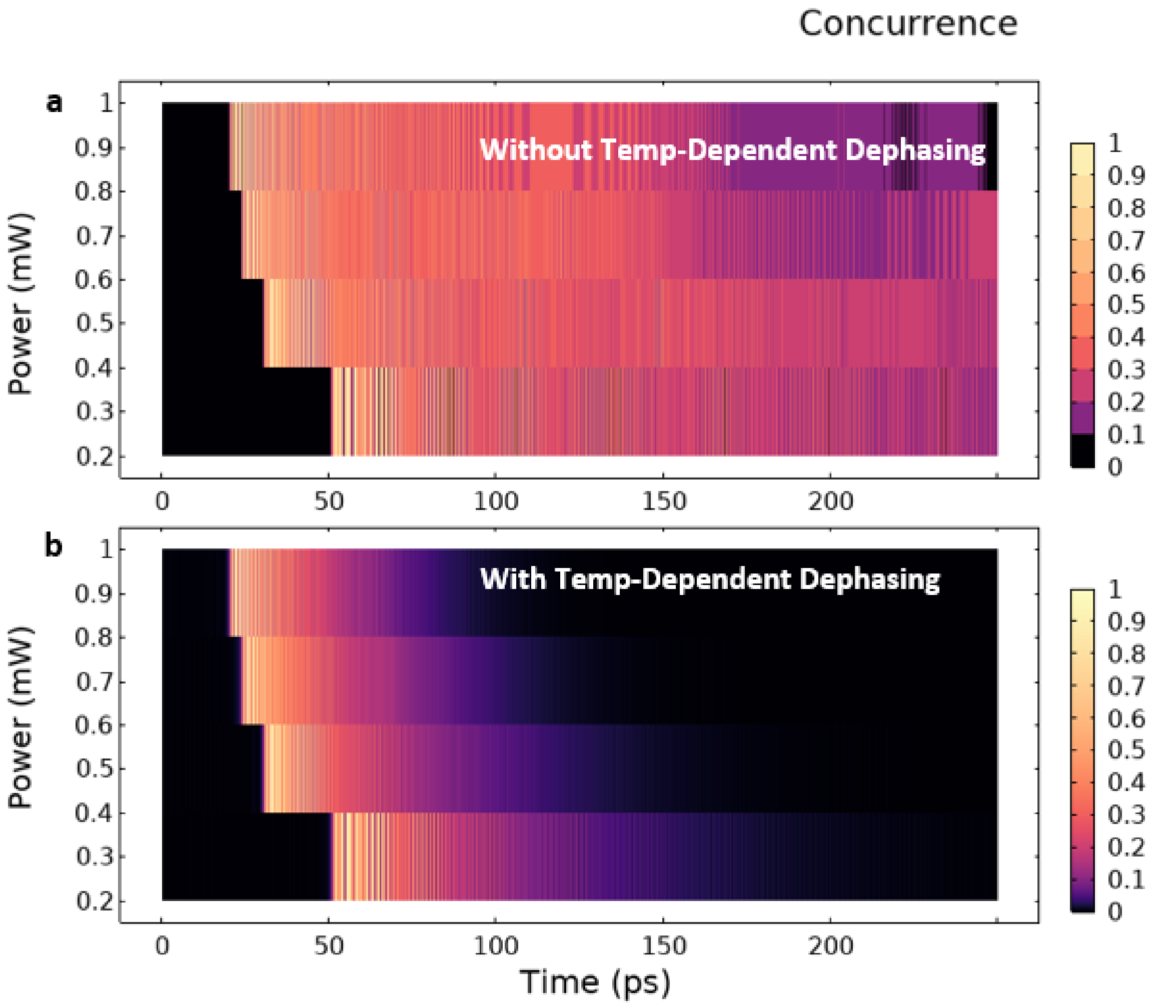

Fig S6. (a) Concurrence is shown in the case when initial cryogenic temperature is set to 30 K, however temperature-dependent dephasing is neglected, leaving only radiative dephasing, in order to demonstrate its contribution to error budgets and reducing its value over time. A clear extension to the period that concurrence is well above 0 can be seen when comparing to the case shown in **(b),** where temperature-dependent dephasing is now included as shown in Fig. 2c of the main text. Here, concurrence tends towards 0 over much shortened time periods as temperatures approach 50 K. See Figure S5 for the change in temperature over time.

REFERENCES

(1) COMSOL Multiphysics® v. 6.1. www.comsol.com. COMSOL AB, S., Sweden.
(2) Abadía, N.; Bello, F.; Zhong, C.; Flanigan, P.; McCloskey, D. M.; Wolf, C.; Krichevsky, A.; Wolf, D.; Zong, F.; Samani, A.; et al. Optical and thermal analysis of the light-heat conversion process employing an antenna-based hybrid plasmonic waveguide for HAMR. *Opt. Express* **2018**, *26* (2), 1752-1765.
(3) Krichevsky, A.; Bello, F.; Wolf, C.; Zong, F.; Wolf, D.; Mccloskey, D.; Ballantine, K.; Donegan, J., Architecture for metal-insulator-metal near-field transducer for heat-assisted magnetic recording. US Patent 10249336, **2019**.
(4) Tian, K.; Xia, J.; Elgammal, K.; Schöner, A.; Kaplan, W.; Karhu, R.; Ul-Hassan, J.; Hallén, A. Modelling the static on-state current voltage characteristics for a 10 kV 4H–SiC PiN diode. *Materials Science in Semiconductor Processing* **2020**, *115*, 105097.
(5) Bello, F.; Kongsuwan, N.; Donegan, J. F.; Hess, O. Controlled Cavity-Free, Single-Photon Emission and Bipartite Entanglement of Near-Field-Excited Quantum Emitters. *Nano Letters* **2020**, *20* (8), 5830-5836. DOI: 10.1021/acs.nanolett.0c01705.
(6) Bello, F.; Sanvito, S.; Hess, O.; Donegan, J. F. Shaping and Storing Magnetic Data Using Pulsed Plasmonic Nanoheating and Spin-Transfer Torque. *ACS Photonics* **2019**, *6* (6), 1524-1532.
(7) Deinega, A.; Seideman, T. Self-interaction-free approaches for self-consistent solution of the Maxwell-Liouville equations. *Physical Review A* **2014**, *89* (2), 022501.
(8) Ekici, O.; Harrison, R. K.; Durr, N. J.; Eversole, D. S.; Lee, M.; Ben-Yakar, A. Thermal analysis of gold nanorods heated with femtosecond laser pulses. *Journal of Physics D: Applied Physics* **2008**, *41* (18), 185501.
(9) Morioka, N.; Babin, C.; Nagy, R.; Gediz, I.; Hesselmeier, E.; Liu, D.; Joliffe, M.; Niethammer, M.; Dasari, D.; Vorobyov, V.; et al. Spin-controlled generation of indistinguishable and distinguishable photons from silicon vacancy centres in silicon carbide. *Nature Communications* **2020**, *11* (1), 2516.
(10) Udvarhelyi, P.; Thiering, G.; Morioka, N.; Babin, C.; Kaiser, F.; Lukin, D.; Ohshima, T.; Ul-Hassan, J.; Son, N. T.; Vučković, J.; et al. Vibronic States and Their Effect on the Temperature and Strain Dependence of Silicon-Vacancy Qubits in 4H-SiC. *Physical Review Applied* **2020**, *13* (5), 054017.
(11) Jahnke, K. D.; Sipahigil, A.; Binder, J. M.; Doherty, M. W.; Metsch, M.; Rogers, L. J.; Manson, N. B.; Lukin, M. D.; Jelezko, F. Electron–phonon processes of the silicon-vacancy centre in diamond. *New Journal of Physics* **2015**, *17* (4), 043011.
(12) Wein, S. C.; Loredo, J. C.; Maffei, M.; Hilaire, P.; Harouri, A.; Somaschi, N.; Lemaître, A.; Sagnes, I.; Lanco, L.; Krebs, O.; et al. Photon-number entanglement generated by sequential excitation of a two-level atom. *Nature Photonics* **2022**, *16* (5), 374-379.
(13) Finco, G.; Miserocchi, F.; Maeder, A.; Kellner, J.; Sabatti, A.; Chapman, R. J.; Grange, R. Time-bin entangled Bell state generation and tomography on thin-film lithium niobate. *npj Quantum Information* **2024**, *10* (1), 135.
(14) Biswas, T.; García Díaz, M.; Winter, A. Interferometric visibility and coherence. *Proceedings of the Royal Society A: Mathematical, Physical and Engineering Sciences* **2017**, *473* (2203), 20170170.

(15) Wootters, W. K. Entanglement of Formation of an Arbitrary State of Two Qubits. *Physical Review Letters* **1998**, *80* (10), 2245-2248.
(16) Cygorek, M.; Ungar, F.; Seidelmann, T.; Barth, A. M.; Vagov, A.; Axt, V. M.; Kuhn, T. Comparison of different concurrences characterizing photon pairs generated in the biexciton cascade in quantum dots coupled to microcavities. *Physical Review B* **2018**, *98* (4), 045303.
(17) Becker, J. N.; Becher, C. Coherence Properties and Quantum Control of Silicon Vacancy Color Centers in Diamond (Phys. Status Solidi A 11⁄2017). *physica status solidi (a)* **2017**, *214* (11), 1770170.
(18) Hashemi, A.; Linderälv, C.; Krasheninnikov, A. V.; Ala-Nissila, T.; Erhart, P.; Komsa, H.-P. Photoluminescence line shapes for color centers in silicon carbide from density functional theory calculations. *Physical Review B* **2021**, *103* (12), 125203.
(19) Bathen, M. E.; Galeckas, A.; Karsthof, R.; Delteil, A.; Sallet, V.; Kuznetsov, A. Y.; Vines, L. Resolving Jahn-Teller induced vibronic fine structure of silicon vacancy quantum emission in silicon carbide. *Physical Review B* **2021**, *104* (4), 045120.
